# Potential Role of Protein Disulfide Isomerase in Metabolic Syndrome-Derived Platelet Hyperactivity

**DOI:** 10.1155/2016/2423547

**Published:** 2016-12-07

**Authors:** Renato Simões Gaspar, Andrés Trostchansky, Antonio Marcus de Andrade Paes

**Affiliations:** ^1^Laboratory of Experimental Physiology, Department of Physiological Sciences, Federal University of Maranhão, São Luís, MA, Brazil; ^2^Departamento de Bioquímica and Center for Free Radical and Biomedical Research, Facultad de Medicina, Universidad de la República, Montevideo, Uruguay; ^3^Health Sciences Graduate Program, Biological and Health Sciences Center, Federal University of Maranhão, São Luís, MA, Brazil

## Abstract

Metabolic Syndrome (MetS) has become a worldwide epidemic, alongside with a high socioeconomic cost, and its diagnostic criteria must include at least three out of the five features: visceral obesity, hypertension, dyslipidemia, insulin resistance, and high fasting glucose levels. MetS shows an increased oxidative stress associated with platelet hyperactivation, an essential component for thrombus formation and ischemic events in MetS patients. Platelet aggregation is governed by the peroxide tone and the activity of Protein Disulfide Isomerase (PDI) at the cell membrane. PDI redox active sites present active cysteine residues that can be susceptible to changes in plasma oxidative state, as observed in MetS. However, there is a lack of knowledge about the relationship between PDI and platelet hyperactivation under MetS and its metabolic features, in spite of PDI being a mediator of important pathways implicated in MetS-induced platelet hyperactivation, such as insulin resistance and nitric oxide dysfunction. Thus, the aim of this review is to analyze data available in the literature as an attempt to support a possible role for PDI in MetS-induced platelet hyperactivation.

## 1. Introduction

Definition of Metabolic Syndrome (MetS) has been a matter of intense scientific output over the last decades, reaching a consensus by The National Cholesterol Education Program–Adult Treatment Panel (NCEP ATP III) to include five major features: visceral obesity, hypertension, dyslipidemia, insulin resistance, and high fasting glucose levels. Diagnostic criteria for MetS must include at least three out of these five features [1]. MetS has become a worldwide epidemic, alongside with a high socioeconomic cost whose prevalence widely ranges from 8% to 43% in men and 7% to 56% in women [2–4]. Importantly, the presence of MetS is associated with a substantial 5-fold increased risk of developing diabetes mellitus (DM) and a 2-fold increase in the development of cardiovascular disease, concurring for higher likelihood to suffer ischemic events [2–5]. In fact, MetS is an independent risk factor for cardiovascular disease (CVD), leading patients to exhibit a prothrombotic and proinflammatory status [6, 7]. As a long-term outcome, MetS individuals tend to develop atherosclerotic plaque as a chronic inflammatory process characterized by increased levels of inflammatory markers, such as tumor necrosis factor *α*, interleukin-6, leptin, angiotensin II, and plasminogen activator factor 1, all of them capital prothrombotic factors [6, 7].

Besides increased inflammatory markers, the prothrombotic state in MetS is mainly caused by endothelial dysfunction and platelet hyperactivity. Both lipotoxicity and insulin resistance contribute to increased oxidative stress (OxS) in the endothelium, leading to enhanced production of reactive oxygen species (ROS) by various isoforms of NADPH oxidase (Nox) and reduced nitric oxide (NO) production and bioavailability, consequent to lower expression and/or uncoupling of endothelial nitric oxide synthase (eNOS) as well as increased reactivity with superoxide (O_2_^∙−^) [8]. Platelets are key players involved in pathologic thrombosis through increased adhesion to the compromised endothelium, being also affected by the increased OxS present in MetS [9, 10]. It has been shown that MetS subjects have increased mean platelet volume, an independent predictor of vascular events [11]. Moreover, there is an increase of proaggregatory and prothrombotic mediators such as thromboxane A_2 _(TxA_2_) and adhesion molecules such as P-selectin, while inhibitory components, like NO, are decreased [12]. Overall, there is an increase in prothrombotic factors with a concomitant decrease in inhibitory components in both endothelium and platelets that concur for increased CVD in MetS.

The Protein Disulfide Isomerase (PDI) is a family of thiol isomerases originally found in the endoplasmic reticulum (ER) that were later discovered in the cytosol and surface of endothelial cells and platelets, among others [13, 14]. The most abundant and physiologically relevant member is PDIA1, the product of the* P4HB* gene, with a molecular weight of 57,000 Da and five subunits: four thioredoxin-like domains (a-b-b′-a′), one C-terminal extension domain, besides one x-linker sequence between b′ and a′ [15, 16]. PDIA1 is also an important regulator of thrombus formation, rapidly binding to *β*_3_ integrins on the endothelium upon injury [17]. In platelets, membrane PDI members, such as PDIA1, ERP5, and ERP57, are known for their paramount importance in platelet aggregation through the isomerization of a disulfide bond in the *α*_2b_ *β*_3_ integrin, which is the final convergent pathway in virtually all mechanisms of platelet aggregation [18]. In addition, platelet surface PDI participates in platelet adhesion through a close interaction with collagen receptor *α*_2_ *β*_1_ [19], GP1b*α* [20], vitronectin [21], and thrombospondin 1 [22]. Despite the already established importance of PDI proteins, precise mechanisms through which surface thiol isomerases interact with integrins and other platelet membrane receptors are still unclear. Since MetS involves many risk factors associated to changes in the coagulation pathway, the aim of this review is to analyze the potential role of platelet surface PDIA1, henceforth referred as PDI, as a central player in platelet hyperactivation under MetS.

## 2. Metabolic Syndrome and Vascular Oxidative Stress

ROS, specially O_2_^∙−^ and hydrogen peroxide (H_2_O_2_), are ubiquitous oxidants of moderate reactivity and brief half-life found in virtually all biological systems as byproducts of oxygen metabolism [23]. At low levels, ROS are key players in many biochemical processes, such as signaling cascades, gene transcription, cellular growth and migration, and apoptosis [24]. In particular at the vascular system, ROS participate in controlling vasodilation and platelet adherence/aggregation [23]. However, when ROS generation is excessive and not compartmentalized, exceeding endogenous antioxidant capacity, cells and tissues progress to OxS, which is considered an early event in the pathophysiology of most chronic noncommunicable diseases associated to MetS [25, 26].

The vascular OxS observed in MetS leads to a change in plasma redox state, inciting a prooxidant environment due to the imbalance of two central thiol/disulfide couples, glutathione/glutathione disulfide (GSH/GSSG), and cysteine/cystine (Cys/CySS) [25, 27]. The reduced partners (GSH or Cys) help in maintaining the thiol/disulfide redox state in proteins, as well as the redox state of ascorbate and vitamin E in their reduced healthy forms by their participation in peroxides removal. Under prooxidant conditions, GSH levels decline in both intracellular and extracellular environment of vascular cells in parallel with an increase in GSSG. Thus, measurement of reduced and oxidized products, as well as their ratios, can provide a useful indicator of OxS in human plasma [25]. Several thiol-containing proteins at the surface of vascular cells, such as thioredoxin and its relatives from PDI family, in response to variable concentrations of ROS, alter the redox state of critical thiols that leads to ROS-driven cellular activation [28]. Noteworthy, recent reports have corroborated the impact of MetS on plasma redox state, with particular emphasis on the assessment of redox status as a tool to predict different outcomes in prediabetic patients [29, 30].

The phagocytic and nonphagocytic isoforms of Noxes are the primary source of ROS and have been consistently implicated in different vascular pathologies [31, 32]. Nox complexes are composed of multiple subunits comprising catalytic (Nox 1–5) and regulatory (p22^phox^, p40^phox^, p47^phox^, p67^phox^, Noxo 1, Noxa 1, and the small GTPases Rac 1 and Rac 2) components, whose expression may vary according to the cell type [33]. Nox4 is associated to cell differentiation of vascular smooth muscle cells [34], whereas Nox1 supports cellular proliferation and migration [35]. Endothelial cells express four Nox isoforms (Nox1, Nox2, Nox4, and Nox5), from which Nox4 is the most highly expressed, and promote H_2_O_2_-derived endothelium preservative actions [36, 37]. On the other hand, expression levels of the other isoforms have been directly implicated in endothelial dysfunction [38].

In platelets, Nox2 was identified by the localization of membrane p22^phox^, cytosolic p47^phox^ subunits, and more recently the catalytic gp91^phox^ subunit [39, 40]. Similarly, Nox1 is also expressed in human platelets, although in a lesser extent when compared to Nox2 [41]. The same study failed to localize Nox4 and Nox5 in platelets, even though further studies are needed to address this matter. Since platelets express Nox1 and Nox2, Delaney and colleagues [42] compared the differential roles of these enzymes in platelet activation and thrombosis. They showed that Nox2, but not Nox1, is required for thrombus formation, whereas none of the enzymes altered tail bleeding time in mice, suggesting further studies should focus on whether Nox-dependent ROS generation may become a potential antithrombotic target without significant bleeding complications [42].

In addition to Nox enzymes, there are different nonenzymatic and enzymatic pathways involved in the formation of ROS in vascular milieu, among them, spontaneous dysmutation of oxygen, leakage of the mitochondrial electron transport chain, myeloperoxidase, xanthine oxidase, cyclooxygenases, and uncoupled NOS [43]. Virtually all these mechanisms may concur for MetS-associated cellular damage, which leads to increased formation of advanced glycation end products (AGE) and its receptors, hexosamine pathway overactivity, increased polyol pathway flux, activation of protein kinase C isoforms [44, 45], lipotoxicity [46], and increased inflammatory profile [47]. Therefore, endothelial cells and platelets from MetS patients suffer from this marked increase in ROS generation, playing a pivotal role in the macro- and microvascular complications of this syndrome.

## 3. Metabolic Syndrome and Platelet Hyperactivity

The damage caused by OxS has been shown to increase platelet aggregation in MetS subjects and decrease aspirin response in DM [6, 10, 48]. These can be explained by several mechanisms: increased platelet secretion of TxA_2_ and prostaglandins (PG), decreased expression of NOS in both endothelium and platelets in addition to a decreased production of prostacyclin (PGI_2_) at the endothelium; decreased platelet response to NO and platelet insulin resistance. Among these, the development of insulin resistance and the impairment of NO homeostasis are arguably the most substantial pathways involved in platelet hyperactivation in MetS.

### 3.1. Thromboxane and F2-Isoprostanes Overproduction in Platelets

TxA_2_ is one of the byproducts of arachidonic acid (AA) oxidation by prostaglandin endoperoxide H_2 _synthase-1 (PGHS-1), also known as cyclooxygenase-1 (COX-1), in platelets [49]. TxA_2_ is synthesized by platelets and acts as an agonist in platelet aggregation and activation, through the ligation of its own G protein-coupled receptor, leading to increased *α*_2b_ *β*_3_ expression, the latter being blocked by the COX-inhibitor aspirin [50]. Besides TxA_2_, F2-isoprostanes are also derived from AA oxidation, stimulating platelet aggregation and complementing TxA_2_ actions. Specifically, 8-iso-PGF2*α* is secreted by platelets upon stimulus, enhancing platelet activation and adhesive reactions to other agonists at low concentrations, through interaction with thromboxane receptor [51, 52]. Noteworthy, urinary secretion of 8-iso-PGF2*α* is also considered a clinical marker of platelet activity [52], which has been found to be increased in obese women [53].

Increased platelet ROS formation in MetS overactivates platelet Nox2 partly through oxidized low-density lipoprotein (oxLDL) ligation of platelet CD36 [54], causing an increase in cytosolic peroxide tone, that is, increased peroxynitrite generation, that subsequently stimulates COX-1 activity [49, 55]. This setting enhances TxA_2_ and 8-iso-PGF2*α* levels through lipid peroxidation and redox-catalyzed conversion of AA into F2-isoprostanes [52]. Interestingly, it seems platelet Nox2 is an important regulator of 8-iso-PGF2*α*, since chemical or hereditary inhibition of Nox2 strongly decreases 8-iso-PGF2*α* generation in platelets [39]. Since COX-1 activity is based in the continuous generation of a lipid-derived radical, besides the reductant environment of the platelet, TxA_2_ pathway is continuously interrupted and requires a permanent reactivation by peroxides [49]. Therefore, OxS can exacerbate platelet aggregation in MetS by changing the intracellular peroxide and peroxynitrite levels, culminating in TxA_2_ and F2-isoprostanes overproduction, a mechanism at least partially regulated by Nox2.

### 3.2. Dysfunctional NO Effects in Platelets

NO is a potent vasodilator and antiplatelet mediator whose bioavailability is inversely correlated with cardiovascular risk [56–59]. Under normal conditions, NO derived from endothelial and platelet NOS diffuses toward circulating platelets in order to activate guanylate cyclase (GC), thus augmenting cyclic guanosine monophosphate (cGMP) levels. Increased levels of cGMP as well as cyclic adenosine monophosphate (cAMP) induce the phosphorylation of vasodilator-stimulated phosphoprotein (VASP) that will inactivate integrin *α*_2b_ *β*_3_ [60–62]. NO also decreases intracellular Ca^2+^ levels [63], inhibits thromboxane receptors in platelets [64], and diminishes platelet recruitment in thrombus formation [65]. Furthermore, in vascular smooth muscle cells, NO is a potent vasodilator that reduces intracellular Ca^2+^ levels by the abovementioned mechanisms [66].

However, in the context of increased OxS induced by MetS or aging, intraplatelet ROS overproduction decreases NO bioavailability by forming reactive nitrogen species (RNS), such as peroxynitrite, leading to platelet hyperactivation [61, 67]. Of note, peroxynitrite induces platelet aggregation with increased intracellular Ca^2+^ concentration [68], while it also oxidizes several proteins that blunt NOS and reduce platelet antioxidant capacity [58, 69]. Platelet NOS was found to be downregulated in MetS patients, which could partially explain the decreased NO production in these subjects when compared to healthy ones [70]. Besides compromised NO bioavailability, platelets from patients with unstable coronary syndrome showed impaired antiplatelet response to the NO donor sodium nitroprusside, suggesting a platelet NO resistance that could be associated to increased OxS [71]. Thus, OxS causes not only a decrease in platelet NO bioavailability, but also a dysfunctional response to its action.

### 3.3. Dysfunctional Insulin Effects in Platelets

Since the discovery of insulin receptors in human platelets, insulin signaling has been considered an important regulator of its function. Hajek and colleagues were the first to demonstrate that platelets possess insulin receptors, with a density of roughly 500 receptors/cell, comparable to other insulin-sensitive cell types [72]. Physiologically, insulin binds to its membrane receptor, provoking the autophosphorylation of its *β*-chain and activating the classical insulin's signaling pathway [73]. In fact, it has been shown that insulin inhibits platelet aggregation in healthy nonobese subjects [74, 75], by a mechanism involving inhibition of tissue factor (TF) and modulation of plasminogen activator inhibitor-1 (PAI-1) concentrations [69]. Moreover, other groups reported that insulin decreases intraplatelet Ca^2+^ content [76] and reduces platelets' response to agonists possibly due to the activation of eNOS [77] and sensitization of platelets to the inhibitory effects of NO [12, 78].

Similar to NO dysfunctional effects in MetS, it has been shown that obese DM subjects blunted insulin's antiplatelet effects, confirming that human platelets can undergo insulin resistance. Insulin resistance is defined by the lack of insulin's actions in platelets, which downregulates IRS-1/Akt pathway, culminating in elevated intracellular Ca^2+^ content and proaggregatory mediators. In fact, platelets from diabetic patients exhibit faster and higher aggregation when compared to healthy ones [69, 79]. Moreover, insulin resistance augments intraplatelet synthesis of PAI-1 and secretion of thromboxane metabolites, thus creating a proaggregatory environment [69]. Finally, there is increased thrombin and fibrin generation, with a prothrombotic fibrin clot phenotype in diabetic patients [80]. Thus, platelet insulin resistance is one of the main contributors to OxS-derived platelet hyperactivation in MetS, even though there is no pathophysiological model to explain how platelets become insulin resistant.

## 4. Protein Disulfide Isomerase and Platelet Hyperactivation in MetS

**Figure 1:**
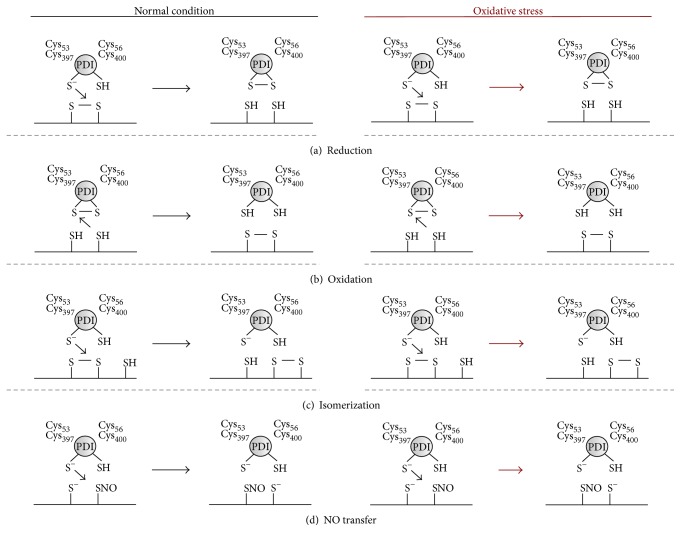
Reactions catalyzed by PDI and possible unbalance in oxidative stress. In (a), PDI catalyzes reduction of a disulfide bond to a dithiol through an attack from its thiolate anion located in Cys_53_ or Cys_397_, whereas in (b) shows PDI oxidizing a dithiol into a disulfide bond. In (c), PDI isomerizes a disulfide bond in the same molecule. These reactions are expected to be increased in OxS, since the reduction of disulfide bonds has been shown to promote platelet aggregation and isomerization is an essential step towards *α*_2b_ *β*_3_ activation [18]. In (d), PDI reacts with NO to promote transnitrosation, shifting NO from one molecule to another or within the same molecule. It should be noted that PDI might also catalyze denitrosation, releasing NO from S-nitrosothiols. This reaction is expected to be decreased in oxidative stress mainly due to the decreased NO bioavailability.

PDI is an ubiquitous chaperone, structurally divided in five subunits: four thioredoxin-like domains (a-b-b′-a′) and one C-terminal extension domain, besides one x-linker sequence between b′ and a′ [15, 16]. Among these, its catalytic redox motif CGHC is present in both a and a′ domains in a constant balance between disulfide and dithiol forms. These CGHC motives confer PDI its ability to catalyze oxidation, reduction, and isomerization reactions (Figure 1), through redox exchanges apparently guided by a trial and error process [16]. Even though containing a C-terminal KDEL ER-retention sequence, PDI is also found in cytosol and surface membrane of numerous cell types, including platelets [14]. Interestingly, besides surface membrane, platelet PDI was localized in the sarco-/endoplasmic reticulum, being mobilized to the surface during platelet activation through a mechanism requiring actin polymerization [81]. Important to this review, we refer specifically to platelet surface PDI.

In platelets, PDI is known for its paramount importance in platelet aggregation through the isomerization of a disulfide bond in the *α*_2b_ *β*_3_ integrin [18]. Such integrin is considered the most important component and final convergent pathway in virtually all mechanisms of platelet aggregation [18]. In fact, anti-PDI antibody inhibits platelet aggregation [82], whereas the addition of reduced PDI prior to agonists enhances maximum aggregation [83]. Of note, it has been recently showed that the C-terminal CGHC motif of PDI is essential for its function in thrombus formation and platelet aggregation [83]. Moreover, PDI is also implicated in the function of other integrins, such as the collagen receptor *α*_2_ *β*_1 _[19] and the von Willebrand factor receptor glycoprotein 1b*α* [20], even though the precise mechanism of such interactions is unclear. Overall, PDI is considered a prothrombotic enzyme, directly implicated in platelet activation, aggregation, and adhesion.

Strikingly, PDI seems to be related to platelet insulin resistance and consequent hyperactivity in OxS induced by MetS. Contrasting with insulin's TF inhibition, PDI has been described as an essential component of TF activation. A proposed working model states that reduced PDI, secreted by activated platelets, reacts with low procoagulant activity TF to yield a TF with high procoagulant activity through the formation of a disulfide bond between Cys_186_ and Cys_209_ residues on TF molecule. Additionally, PDI may promote fibrin generation [84]. Overall, this suggests that while insulin inhibits TF activation, PDI works on the opposite side by augmenting TF procoagulant activity and increasing fibrin generation upon injury. Nonetheless, insulin resistance and PDI seem to exert similar effects on platelet activation. Even though no study has ever demonstrated whether PDI can desensitize platelet's insulin receptors, the likewise effects of insulin resistance and PDI on platelet function could lead to a possible connection between these two factors.

**Figure 2:**
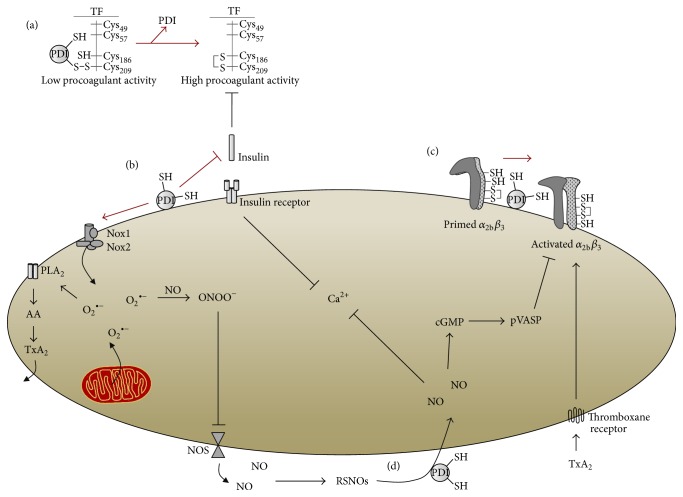
PDI participates in mechanisms of platelet hyperactivation induced by metabolic syndrome. In (a), PDI promotes the procoagulant activity of tissue factor (TF) through the formation of a disulfide bond between TF Cys_186_ and Cys_209_. In (b), PDI inhibits insulin's action by reducing a disulfide bond that precipitates insulin's *β*-chain, preventing insulin's inhibitory activity upon TF and insulin's intracellular signaling in platelets. Also, PDI regulates Nox enzymes, promoting stronger generation of O_2_^∙−^ that can either react with NO, forming peroxynitrite that will inhibit nitric oxide synthase (NOS) or induce thromboxane (e.g., A_2_) generation through phospholipase A_2_ (PLA_2_) and subsequent COX-derived arachidonic acid (AA) platelet metabolism. (c) PDI promotes the isomerization of a disulfide bond in *α*_2b_ *β*_3_ integrin. Finally, in (d), PDI has a paradoxical effect in platelet aggregation, acting as a nitric oxide (NO) carrier and releaser through transnitrosation and denitrosation reactions of S-nitrosothiols (RSNOs). Arrows in red indicate overactivated mechanisms.

A plausible hypothesis is that PDI's procoagulant reactions could be increased in insulin resistance and OxS in detriment of decreased insulin activity or even that insulin resistance could be, at least in part, accounted for increased PDI activity. This is supported by the well-characterized in vitro reaction between PDI and insulin, where the first reduces a disulfide bond in the latter, causing the precipitation of insulin's *β*-chain [85]. In vivo, it has been reported that DM patients release more platelet-derived microparticles (pMPs) [86]. Importantly, pMPs contain catalytically active PDI, and DM subjects have increased levels of PDI-containing pMPs [87]. In fact, plasma samples from DM patients present roughly 50% more pMPs than healthy subjects, also exhibiting 60% more PDI and 70% more PDI activity [87]. Noteworthy, these microparticles were able to catalyze insulin disulfide reduction, abrogating insulin's activity, as shown by loss of Akt phosphorylation in 3T3-L1 cells [87]. Therefore, it is reasonable to suggest that the increased PDI secretion from DM platelets reduces insulin's bioavailability, contributing to the lack of insulin's action found in MetS platelets (Figure 2). Nonetheless, it should be stressed that further studies are needed to address whether PDI is an important cue in MetS-induced platelet hyperactivity, specifically if secreted platelet PDI is able to desensitize insulin receptor in various cell types.

PDI is also involved in platelet NO homeostasis [88], providing evidence for a paradoxical effect of PDI in platelet activation. Previous studies have shown that PDI acts as an NO carrier through vascular cells by transnitrosation reactions, exchanging the nitrosonium ions between cysteines (Figure 1(d)) [89, 90]. Likewise, it was also shown that platelet PDI denitrosates S-nitrosothiols (RSNOs), thus releasing NO and increasing its bioavailability [91]. Moreover, RSNOs seem to be denitrosated by the same CGHC active site that gives PDI its proaggregatory properties [83, 91]. These findings were further supported by Bell et al. [92] that showed PDI is implicated in a wide range of NO-related signals and not only with RSNOs, as previously thought. However, NO can also attack PDI in an S-nitrosylation reaction, which transfers NO to critical cysteines in CGHC active sites. Such reaction inhibits PDI isomerization and chaperone activities by roughly 50%, which could in turn compromise the aforementioned mechanisms of platelet aggregation through *α*_2b_ *β*_3_ [93]. Despite acting as a NO donor, PDI paradoxically inhibits NO effects in vascular smooth cells by a thiol-disulfide exchange between PDI's CGHC active site and the *α* or *β* domains of soluble GC [94, 95]. Therefore, platelet PDI improves NO bioavailability, acts as an NO carrier, while it can also be inhibited by NO itself, whereas in vascular smooth cells PDI abrogates NO effects.

Nevertheless, it is important to notice that the abovementioned studies took place under physiological conditions and the interaction between PDI and NO was not tested under increased OxS, nor was it tested in MetS subjects. One could hypothesize that increased ROS production, causing cellular damage and increased OxS, coupled with alterations in plasma GSH/GSSG and Cys/CySS could interfere with PDI's denitrosation activity or even revert its effect. Additionally, decreased NO bioavailability due to peroxynitrite formation leads to lower extents of PDI reacting with available NO and/or less NO inhibiting PDI isomerase activity, which would shift the enzyme's activity to proaggregatory pathways (Figure 2). These hypotheses are based on the well-established decrease of NO bioavailability in MetS (detailed in Section 3.2) and should be addressed in future studies, since dysfunctional NO effect on platelets is an important cue to better understand platelet hyperactivity. Nonetheless, studies are needed to investigate whether there is a link between PDI and decreased NO bioavailability or diminished platelet NO response, given that this protein is of capital importance to platelet function.

Last but not least, it has been suggested that PDI act as a modulator of distinct members of Nox enzymes in the vascular system [96]. There is a close association between PDI and Nox1 [96–98], phagocytic Nox2 [99, 100], and Nox4 [97, 101], which has been demonstrated through biochemical and molecular approaches of gene silencing and overexpression. Specifically to Nox2, PDI regulates its function possibly through mechanisms involving thiol groups on its various subunits and therefore contribute to ROS generation [96]. Early studies have found increased protein expression and activity of Nox2 subunits p22^phox^ and gp91^phox^ in pMPs from septic patients [102], which were later demonstrated also to present higher levels of PDI, as well [103]. Even though there is no study addressing such interaction in platelets from MetS patients, it is plausible to infer that PDI might also regulate Nox2 activity in these cells, further contributing for OxS-driven NOS uncoupling, thromboxane generation as well as insulin resistance.

## 5. Conclusions

MetS increases cardiovascular risk and mortality, being considered a worldwide epidemic. Among the cardiovascular outcomes implicated in MetS, platelet hyperactivation plays a pivotal role in morbidity and mortality. At the same time, PDI is an important regulator of platelet function. However, to the best of our knowledge, no study has investigated the likely contribution of PDI in MetS-induced platelet hyperactivity, nor has it ever been proposed. Therefore, we propose that PDI could be a potential culprit of MetS-induced platelet hyperactivity, possibly through a deficient PDI denitrosation activity, decreased PDI S-nitrosylation and/or less PDI needed for transnitrosation reactions, an increase in TF activation, and insulin resistance caused by increased quantity and/or activity of secreted platelet PDI. Novel original studies are needed to corroborate or reject this hypothesis.
